# The Effect of Unilateral Adrenalectomy on Transformation of Adrenal Medullary Chromaffin Cells *In Vivo*: A Potential Mechanism of Asthma Pathogenesis

**DOI:** 10.1371/journal.pone.0044586

**Published:** 2012-09-05

**Authors:** Cheng-Ping Hu, Ye-Qiang Zou, Jun-Tao Feng, Xiao-Zhao Li

**Affiliations:** 1 Department of Respiratory Medicine, Xiangya Hospital, Central South University, Changsha, China; 2 Department of Respiratory Medicine, The First People’s Hospital of Changde, Changde, China; Indiana University School of Medicine, United States of America

## Abstract

**Background:**

Decreased epinephrine (EPI) is an important underlying factor of bronchoconstriction in asthma. Exogenous β_2_-adrenergic receptor agonist is one of the preferred options to treat asthma. We previously showed that this phenomenon involved adrenal medullary chromaffin cell (AMCC) transformation to a neuron phenotype. However, the underlying molecular mechanism is not fully understood. To further explore this, an asthmatic model with unilateral adrenalectomy was established in this study.

**Methodology/Principal Findings:**

Thirty-two rats were randomly into four groups (n = 8 each) control rats (controls), unilateral adrenalectomy rats (surgery-control, s-control), asthmatic rats (asthma), unilateral adrenalectomy asthmatic rats (surgery-induced asthma, s-asthma). Asthmatic rats and s-asthmatic rats were sensitized and challenged with ovalbumin (OVA). The pathological changes in adrenal medulla tissues were observed under microscopy. EPI and its rate-limiting enzyme, phenylethanolamine N-methyl transferase (PNMT), were measured. Peripherin, a type III intermediate filament protein, was also detected in each group. The asthmatic rats presented with decreased chromaffin granules and swollen mitochondria in AMCCs, and the s-asthmatic rats presented more serious pathological changes than those in asthmatic rats and s-control rats. The expressions of EPI and PNMT in asthmatic rats were significantly decreased, as compared with levels in controls (P<0.05), and a further decline was observed in s-asthmatic rats (P<0.05). The expression of peripherin was higher in the asthmatic rats than in the controls, and the highest level was found in the s-asthmatic rats (P<0.05).

**Conclusion/Significance:**

Compared with asthmatic rats and s-control rats, the transformation tendency of AMCCs to neurons is more obvious in the s-asthmatic rats. Moreover, this phenotype alteration in the asthmatic rats is accompanied by reduced EPI and PNMT, and increased peripherin expression. This result provides further evidence to support the notion that phenotype alteration of AMCCs contributes to asthma pathogenesis.

## Introduction

Asthma is a complex disorder of unknown etiology, characterized by increased bronchial smooth muscle contraction, airway inflammation and tissue remodeling. Epinephrine (EPI), a β_2_-adrenergic receptor agonist, functions to relax bronchial smooth muscle and is used extensively to treat asthma. Asthma patients have been shown to have decreased levels of endogenous EPI, which may explain why administration of exogenous EPI is such an effective treatment [1], [2]. EPI is secreted by adrenal medullary chromaffin cells (AMCCs), which are derived from the same sympathoadrenal precursor cells that produce neurons. For this reason, AMCCs harbor the potential to transform to the neuron phenotype, and *in vitro* exposure of AMCCs to nerve growth factor (NGF) can produce this effect [3]–[5]. NGF, one of the most important neurotrophic factors involved in neuronal growth, survival, and differentiation, has been clinically detected as increased in asthma patients. Moreover, NGF expression appeared to correlate with the degree of bronchial hyperreactivity; although, the exact mechanism and physiologic implication remained unknown [6]. In our previous studies involving induction of AMCC transformation to neurons in asthmatic rats, we observed decreased EPI and elevated NGF in these animals, and noted that this phenomenon was inhibited by administration of anti-NGF [7]–[9]. Thus, elevated NGF might promote AMCC transformation to neuron, which, in turn, would inhibit the synthesis and secretion of EPI and induced bronchoconstriction in asthma.

Furthermore, our recent study indicated that asthma during rodent pregnancy was able to promote AMCCs to differentiate into neurons in the offspring rat pups and inhibited their ability to synthesize EPI. This result suggested that asthma can affect the development of the adrenal medulla *in utero* [10]. Interestingly, our recent results also showed that when these offspring rats matured, their susceptibility to OVA-induced asthma was increased due to the dysfunctional adrenal medulla (unpublished data). This indicated that damage to the adrenal medulla could induce asthma. Together, these data suggested that transition of AMCCs might be a critical process in asthma pathogenesis. Further investigation is required to confirm this. It is important to note that unilateral adrenalectomy led to the dysfunction of adrenal medulla, as evidenced by significantly reduced EPI [11]. This effect has aggravated the asthma condition. However, it remained unknown whether the reduction in EPI was related to the transition of AMCCs. In the current study, unilateral adrenalectomy was investigated for its effects on the structure and function of AMCCs in asthmatic rats. In addition, we also tested whether AMCC transformation to the neuron phenotype was significantly correlated with development of asthma.

## Results

### Airway Responsiveness to Histamine in Asthmatic Rats

**Figure 1:**
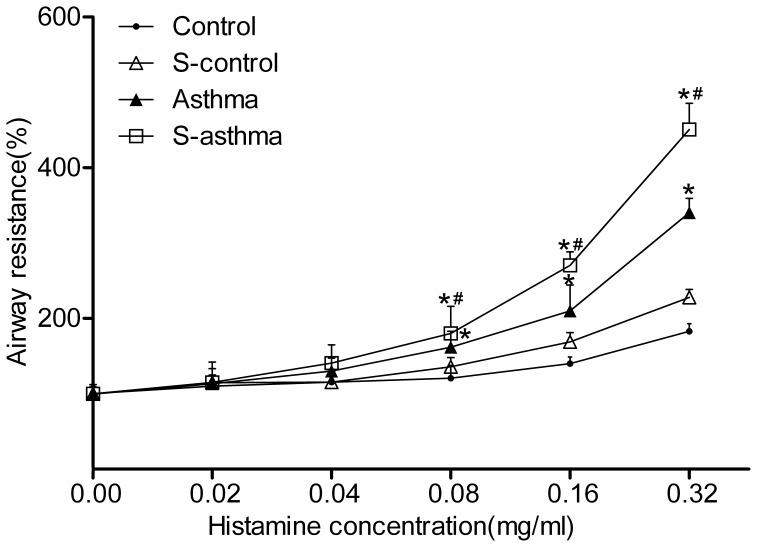
Changes in the airway resistance to histamine in each group. Rats were treated with aerosolized saline or increasing doses of histamine. Airway resistance was calculated as the index of percent increase in airway resistance when compared with the peak of the reaction with baseline. The values were expressed 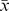
±s (n = 8). * indicates significant difference as compared with the control group (*P*<0.05); ^#^ indicates significant difference between s-asthma and asthma groups (*P*<0.05).

We showed that administration of increasing concentrations of histamine was associated with gradual increases in airway resistance in each group. When the concentration of histamine reached 0.08 mg/mL, the airway responsiveness in asthmatic rats became higher than that in control rats, and this pattern continued for all higher concentrations. Slight increases were found in the s-control rats compared with the control rats, but there were no significant differences between these two groups. Even more extensive increases in airway responsiveness were found in the s-asthmatic rats (Figure 1).

### Morphological Changes of Lung Tissue in Asthmatic Rats

**Figure 2:**
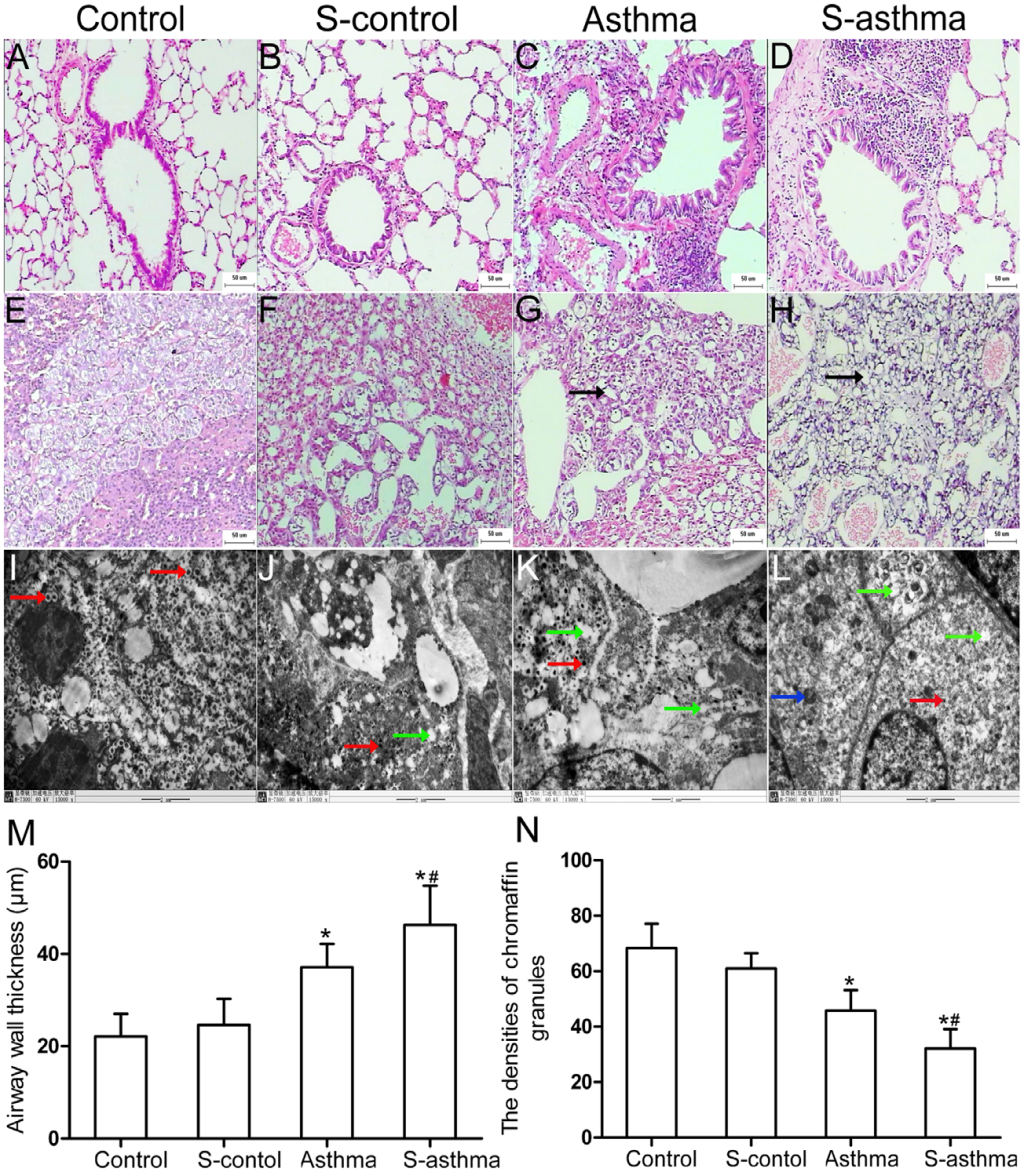
Pathological changes in the lung and adrenal medulla of each group. Representative micrographs (H&E staining, 100×) of lungs from control rats (A), s-control rats (B), asthmatic rats (C), and s-asthmatic rats (D) were shown. Representative micrographs (H&E staining, 100×) of adrenal medulla in control rats (E), s-control rats (F), asthmatic rats (G), and s-asthmatic rats (H) were shown. Representative electron micrographs (15000×) of adrenal medulla in the control rats (I), s-control rats (J), the asthmatic rats (K), and the s-asthmatic rats (L) were presented. Arrows indicate vacuolar degeneration (black), chromaffin granules (red), mitochondria (blue), and the sign of defluxion in chromaffin granules (green). The bar graphs demonstrate the quantitative analysis of the airway wall thickness (M) and the densities of chromaffin granules (N). The values were presented as 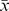
±s (n = 8). * indicates significant difference as compared with the control group (*P*<0.05); ^#^ indicates significant difference between the s-asthma and the asthma groups (*P*<0.05).

We found that, compared with the control rats, the asthmatic rats showed a significantly thicker airway wall (*vs.* control, *P*<0.05), with increased mucosal folding and shedding of epithelial cells. In addition, asthmatic rats had more hypertrophic goblet cells, and the surrounding airway had been infiltrated by eosinophils and lymphocytes. In general, the s-asthmatic rats presented with the same histological findings but the extent was greater, such as in airway wall thickness (*vs.* asthma, *P*<0.05). Compared with the control rats, no obvious changes were found in the s-control rats (Figure 2 A–D and M).

### Alteration of Adrenal Medulla in Each Group

Compared with light microscope findings for the control rats, asthmatic rats had greater amounts of vacuolar degeneration and lipid droplets in AMCCs. Again, a similar but more extensive deterioration was observed in the s-asthmatic rats, which included disordered medullary structure and blood sinus expansion (Figure 2 E–H).

Electron microscope showed a regular morphology of AMCCs in control rats, which was characterized by clear structure of mitochondria and abundant chromaffin granules throughout the cytoplasm. On the contrary, swollen cytoplasm and mitochondria were observed in the s-control rats and the asthmatic rats, and the changes in the asthmatic rats were more serous. Furthermore, the s-asthmatic rats showed similar perturbed changes, but the extent was greater than those in the asthmatic rats. Compared with the control rats, the asthmatic rats presented with significantly decreased densities of chromaffin granules (*vs.* control, *P*<0.05); even lower densities were found in the s-asthmatic rats (*vs.* asthma, *P*<0.05) (Figure 2 I–L and N).

### Phenylethanolamine N-methyl Transferase (PNMT) and Peripherin Expression in Adrenal Medulla of Each Group

**Figure 3:**
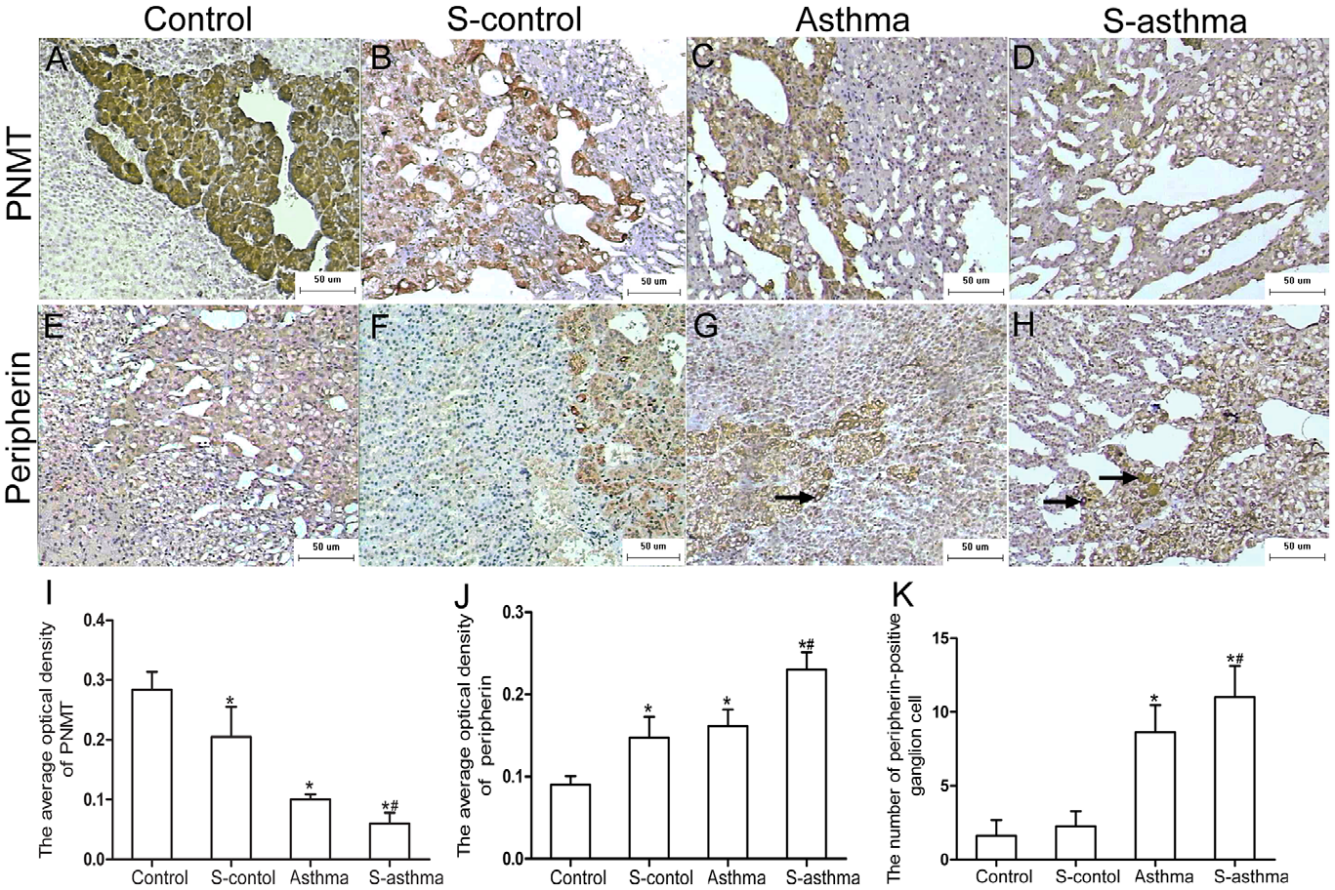
Immunostaining of PNMT and peripherin in adrenal medulla. The expressions of PNMT and peripherin in the adrenal medulla of each group were detected by immunohistochemistry. A representative sample of PNMT immunostaining (DAB, 100×) was presented for the control rats (A), s-control rats (B), the asthmatic rats (C), and the s-asthmatic rats (D). A representative peripherin immunostaining (DAB, 100×) was presented for the control rats (E), s-control rats (F), the asthmatic rats (G), and the s-asthmatic rats (H). Arrows indicate the peripherin-positive ganglion cells. Bar graphs demonstrate the average optical density of PNMT (I) and peripherin (J) and the number of peripherin-positive ganglion cells (K). The values were presented as 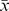
±s (n = 8). * indicates significant difference as compared with the control group (*P*<0.05); ^#^ indicates significant difference between the s-asthma and the asthma groups (*P*<0.05).

Immunostaining of PNMT was mainly localized to the cytoplasm of healthy cells. In control rats, the density of PNMT immunostaining was robust in the adrenal medulla. The PNMT staining was much weaker in s-control rats and asthmatic rats (*vs.* control, all *P*<0.05), indicating that PNMT expression was reduced in these animals. Only a few cells in the s-asthmatic rats stained for PNMT (*vs.* asthma, *P*<0.05) (Figure 3A–D, I).

Immunostaining of peripherin was visualized by a brown coloration mainly located in the membrane and cytoplasm. Compared with the peripherin staining detected in control rats, the density of peripherin was higher in s-control rats and the asthmatic rats (*vs.* control, all *P*<0.05), and was further enhanced in s-asthmatic rats (*vs.* asthma, *P*<0.05) (Figure 3E–H, J). Furthermore, the results indicated that the number of peripherin-positive ganglion cells were increased in the adrenal medulla of asthmatic rats compared with the control rats (*vs.* control, *P*<0.05), and even more in s-asthmatic rats than those in asthmatic rats (*vs.* asthma, *P*<0.05) (Figure 3 E–H and K).

### Peripherin Expression in Adrenal Medulla of Each Group

**Figure 4:**
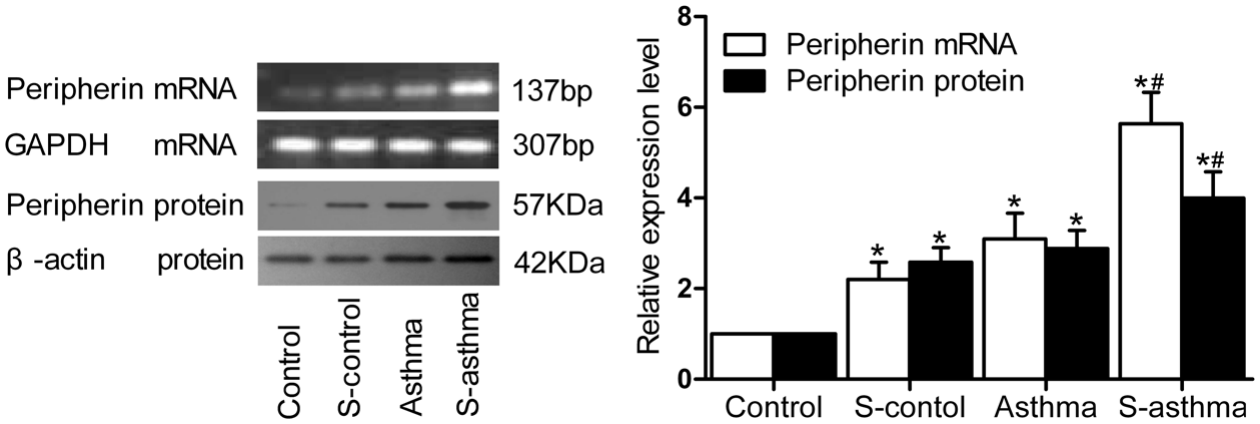
Peripherin expression in adrenal medulla. Total RNA and cellular protein were isolated from adrenal medulla, and expressions of peripherin (mRNA and protein) in each group were detected by RT-PCR and Western blot, respectively. The bar graphs on the right panels demonstrated the densitometry results relative to expression in controls (1.0). The values were expressed as 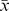
±s. β-actin and GAPDH were used as internal controls. * indicates significant difference as compared with the control group (*P*<0.05); ^#^ indicates significant difference between the s-asthma and the asthma groups (*P*<0.05).

Compared with the control rats, significantly increased peripherin mRNA and protein were found in the s-control rats and the asthmatic rats (all *P*<0.05), as detected by RT-PCR and Western blot, respectively. The s-asthmatic rats had even stronger expressions of both the peripherin mRNA and protein than that in the asthmatic rats (*P*<0.05) (Figure 4).

### EPI Level in Serum of Each Group

Compared with the control rats, the level of serum EPI was slightly decreased in the s-control rats (8.91±1.43 *vs.* 9.54±0.76 ng/mL, *P>*0.05) and significantly decreased in the asthmatic rats (7.28±0.56 *vs.* 9.54±0.76 ng/mL, *P*<0.05). The EPI levels detected in the s-asthmatic rats was significantly lower than that in the asthmatic rats (4.32±0.39 *vs*. 7.28±0.56 ng/mL, *P*<0.05).

### Corticosterone Level in Serum of Each Group

The ELISA results indicated that there was no significant difference in corticosterone levels between the asthmatic rats and the control rats (484.38±26.94 *vs*. 461.12±29.51 pg/mL, *P>*0.05). A similar result was found between the s-control rats and the control rats (479.62±20.28 *vs*. 461.12±29.51 pg/mL, *P>*0.05). However, a remarkable reduction in corticosterone level was observed in the s-asthmatic rats, as compared with their asthmatic counterparts (368.50±19.76 *vs*. 484.38±26.94 pg/mL, *P*<0.05).

### Serum NGF Level in Each Group

Compared with ELISA results from the control rats, serum levels of NGF were increased in the asthmatic rats (89.38±14.40 *vs*. 63.50±13.63 pg/mL, *P*<0.05) and in the s-control rats (84.87±16.96 *vs*. 63.50±13.63 pg/mL, *P*<0.05). Furthermore, NGF level was significantly higher in the s-asthmatic rats than in the asthmatic rats (131.75±16.37 *vs*. 89.38±14.40 pg/mL, *P*<0.05).

## Discussion

The adrenal medulla is a specialized endocrine organ that originates during development from the neuroectoderm at a point when the adrenergic nerves are also arising. Likewise, the AMCCs and sympathetic neurons arise from a common sympathoadrenal precursor cells. The ability of NGF to induce differentiated AMCC to transform into sympathetic neuron is well-documented, and is characterized by observable alterations in morphology and endocrine function. One of the principal changes that mark the phenotypic switch from AMCC to neuron is when EPI, which is one of the principal secretary proteins of AMCCs, is no longer expressed. Interestingly, studies found that glucocorticoid hormones, which antagonize NGF, can block the NGF-induced transformation of AMCCs [3], [4], [12]–[15].

Wang *et al*. reported that the levels of EPI and PNMT (the rate-limiting enzyme of EPI) were commonly decreased in asthmatic rats, whereas NGF expression was increased. Moreover, these asthmatic rats also presented with significant changes in AMCC ultrastructure, including decreased chromaffin granules, and increased lipid. These studies also indicated that NGF-treatment aggravated the AMCC lesions in asthmatic rats, as evidenced by further decreases in EPI and PNMT levels and the numbers of chromaffin granules [7], [8]. These results suggested that there was a tendency towards phenotype conversion from AMCC to neuron in the asthmatic rats and that NGF played an important role in this process.

In the study presented herein, lesions of the adrenal medulla also accompanied asthma onset, and featured vacuolar degeneration, blood sinus expansion, and nucleolemma shrinkage. Furthermore, more serious pathological changes in AMCCs after unilateral adrenalectomy were observed by electron microscopy. Significantly decreased densities of chromaffin granules were observed, accompanied by swollen cytoplasm and mitochondria. These ultrastructural changes (including increased lipids and mitochondrial swelling) were found in chromaffin cells of the asthmatic rats, which were considered as evidence of phenotype switching [10]. A similar result had been reported in another study that showed NGF-induced transdifferentiation in PC12 cells [16]. Both mitochondria and chromaffin granules have high lipid content, which can be modulated by many factors [17]. Significant modulation may trigger the process of cell death (e.g. apoptosis). NGF, the most important growth factor for neuron differentiation, is significantly elevated in asthma. In our study, we also observed elevated peripherin, another marker of neuron differentiation, in AMCCs of asthmatic rats. Thus, the increased lipids and mitochondrial swelling may be considered as the features of AMCC transdifferentiation, rather than apoptosis, in our animal study. Similar to the study reported by Bonini *et al*. [6], we also found that the level of NGF expressed corresponds to the degrees of bronchial hyperreactivity, lung lesions, and AMCC alterations. Collectively, these results supported the notion that the lesions of AMCCs observed in the asthmatic rats were strongly related to the pathogenesis of asthma.

If one of the two glands is removed, the EPI level will decrease. A compensatory mechanism may exist, in which the remaining gland can provide overloaded epinephrine to maintain physiological function; as a result, the decrease in EPI is slight in the s-control group (*vs.* control group, *P>*0.05). However, the compensatory function is limited, and the remaining gland can be impaired easily during the second stress event (s-asthma group *vs.* asthma group, *P*<0.05). In our study, the extent of AMCC alteration that occurred in the remaining gland tissue of the s-asthmatic rats was more remarkable than in the asthmatic rats. In addition, we demonstrated that higher rates of AMCC transition to neuron were accompanied by lower rates of epinephrine secretion. Thus, the aggravated AMCC alteration of the “remaining gland” may have been another mechanism responsible for the decreased epinephrine levels that occurred in the s-asthmatic rats. Certainly, the decreased EPI level is most likely related to PNMT, which is one of the most important rate-limiting enzymes of EPI, and is mainly expressed in AMCCs and not expressed in neurons. Thus, the level of PNMT can be used to assess endocrine function of AMCCs. In this study, we observed a decrease in expression of PNMT in the asthmatic rats, which was even further depressed in response to unilateral adrenalectomy. As a result, the synthesis of EPI is expected to be reduced as well. Indeed, the levels of EPI detected were concomitant with the expression of PNMT in each group.

It has been reported that glucocorticoids can promote the expression of PNMT in adrenal medulla, and that PNMT catalyzes the conversion of norepinephrine to epinephrine [18]–[20]. Some *in vivo* studies have indicated that a sufficiently high concentration of glucocorticoids in the adrenal medulla can prevent fiber outgrowth from medullary chromaffin cells; as such, this process is presumed to contribute, at least partially, to inhibiting spontaneous transformation of adrenal cells into neurons [21]–[23]. To a certain extent, the choice of AMCC fate is determined by the balance of NGF and glucocorticoids present in the milieu at any given time [21], [24]. Thus, the reduced levels of PNMT and EPI in our rats may have been a reflection of the NGF levels, and may have been a principal underlying mechanism guiding the transformation event from AMCC to sympathetic neuron. In other words, the levels of corticosterone might not have been enough to overcome the effects induced by NGF. These data suggest that inhibiting AMCC transformation could be another benefit of supplementing exogenous glucocorticoids in the treatment of asthma patients.

The anti-inflammatory effect is a well-known mechanism of glucocorticoids in the treatment of asthma; therefore, the features of airway inflammation in asthmatic rats and s-asthmatic rats may be related to the decreased corticosteroid levels. In this study, although the corticosterone levels of the asthmatic rats are slightly higher than those of the control rats (*P>*0.05), the extent of inflammation and gland pathological alteration is more serious in the asthmatic group than in the control group. However, it is unlikely that corticosteroid reduction is the only mechanism responsible for asthma pathogenesis; decreases in epinephrine induced by AMCC transdifferentiation might also be associated with this process.

During the development and regeneration of nerve cells, peripherin (a type III intermediate filament) plays an important role in establishing cellular architecture by regulating axon formation [25]. In fact, peripherin-siRNA depleted neurons presented with significantly impaired initiation, extension, and maintenance of neurites [25]. Cells reactive to peripherin only actually constituted a specific sub-population of AMCCs; this idea was supported by the fact that AMCCs, which highly expressed peripherin, had a distinct shape that was resemble to nerve cells [26]. AMCCs were implicated in active down-regulation of the intermediate filament protein that occurred during embryonic development [3]. Consistent with these results, Nie *et al*. found that the expression of peripherin in NGF-treated AMCCs *in vitro* was significantly increased, as compared with that in the control cells by proteomic analysis. This study also showed that asthmatic rats exhibited higher expression of peripherin in adrenal medulla than that the healthy controls, and that these asthmatic rats had AMCC with decreased chromaffin granules [9].

In our asthmatic rats, peripherin expression was also increased, while EPI and PNMT levels were significantly decreased. These expression profiles were markedly aggravated by unilateral adrenalectomy. Moreover, the fact that peripherin-positive ganglion cells presented in asthmatic and s-asthmatic rats, but little or none in control rats, was consistent with our previous report [27]. Increased numbers of ganglion cells generally indicate an alteration of innervations occurring in adrenal medulla, and this phenomenon is considered to be a reflection of neural plasticity. It is possible that this common process is responsible for maintaining the balance of the sympathoadrenomedullary system (affecting both structure and function), and this could be one of the underlying causes for the results. Nonetheless, our experimental results suggested that peripherin was closely related to the transformation from AMCC to neuron.

More severe airway resistance and airway inflammation were found in asthmatic rats with unilateral adrenalectomy than that in the asthmatic rats with intact adrenal medulla, and the further decrease in EPI might be an important reason for this. Unilateral adrenalectomy has been shown to reduce the secretion of hormones, such as EPI and corticosterone [11]. Interestingly, a more apparent switch in phenotype of AMCC was observed in asthmatic rats with unilateral adrenalectomy, as compared with asthmatic rats with intact adrenal medulla, which might explain the further decrease in EPI levels.

Together, this result indicates that unilateral adrenalectomy exacerbates asthma, and this process is strongly associated with AMCC transdifferentiation. These data may explain the physiopathological effects of decreased EPI and consequent bronchoconstriction in asthma, and imply potential mechanisms and involved factors in the pathogenesis of asthma that need to be further determined.

## Materials and Methods

### Experimental Animals and Preparation

All animals used in this study were conventionally-bred 6- to 8-week-old male Sprague-Dawley rats (Experimental Animal Center of Central South University, Changsha, China). This study was carried out in strict accordance with the recommendations from the Guide for the Care and Use of Laboratory Animals published by the National Institutes of Health. The protocol was approved by the Ethics Committee of Asthma Research Institute, Hunan Province (20100606). All surgeries were performed under chloral hydrate anesthesia.

Thirty-two rats were divided among four groups by using a random digits table (*n* = 8 per group): control rats (control), unilateral adrenalectomy rats (surgery-control, s-control), asthmatic rats (asthma), unilateral adrenalectomy asthmatic rats (surgery-induced asthma, s-asthma). The rats were treated according to a method described in a previous study [7]; briefly, on day 0, all animals were anesthetized by intraperitoneal (I.P.) injection of 10% chloral hydrate (350 mg/kg) and the right adrenal gland was excised via back incision from the rats in the s-control group and the s-asthmatic group. The control and asthmatic rats were given the same surgery without removal of the gland. On day 10 and 17, the asthmatic and s-asthmatic rats were sensitized with an I.P. injection of 1 mL sterile saline solution containing 100 mg ovalbumin (OVA) (Sigma, USA), 200 mg aluminum hydroxide (Sigma), and 6×10^9^ heat-killed *Bordetella pertussis* (Wuhan Institute of Biological Products, China). The control rats and the s-control rats were sham-sensitized by I.P. injection of sterile saline containing partes aequales aluminum hydroxide and heat-killed *Bordetella pertussis* without OVA. The sensitized rats were exposed to 1% OVA (wt/vol) aerosol for 30 min every day from day 24 to day 38; the control rats and the s-control rats received aerosol of sterile saline only.

### Measurement of Bronchial Responsiveness

*In vivo* airway responsiveness to histamine was measured at 24 hr after the last OVA challenge using a whole-body plethysmography device (model PLY 3211; Buxco Electronics, USA). Rats were treated for 2 min with aerosolized saline or increasing dosages of histamine generated by an ultrasonic nebulizer, after which airway resistance was measured. Histamine-induced bronchoconstriction was considered as the index of percent increase in airway resistance when the peak of the reaction was compared with baseline airway resistance [27], [28].

### Tissue Preparation

After determination of bronchial responsiveness, all rats were anesthetized using I.P. injection of 10% chloral hydrate (350 mg/kg) prior to the sacrifice. The left adrenal glands were excised and either snap-frozen at −80°C, fixed in 4% paraformaldehyde or fixed in 2% glutaraldehyde, according to the subsequent usages. Serum was obtained from each rat. After the pulmonary artery was perfused, the right middle lung lobe was collected from each rat and fixed in 4% paraformaldehyde.

### Hematoxylin and Eosin (H&E) Staining of Lung and Adrenal Medulla Tissues

Lung and adrenal medulla tissues were collected and fixed in 4% paraformaldehyde, and then embedded in paraffin. Tissue sections (4 µm) were stained with H&E, and the morphological changes were observed under light microscope. The airway wall thickness was quantified by pathological image analysis system.

### Transmission Electron Microscopy

Adrenal medullas were fixed with 2% glutaraldehyde in 0.1 M cacodylate buffer, pH 7.2. Three hours later, specimens were post-fixed in buffered 1% OsO_4_ for 1 hr, following by dehydration in a graded ethanol series and embedding in Epon-Araldite. Ultrathin sections (50 nm) were prepared from the different specimens and stained with uranyl acetate and lead citrate. Examinations of ultrastructure changes were carried out with an H-7500 transmission electron microscope (Hitachi, Japan). All samples were assessed by two pathologists who were blinded to the corresponding treatments. Using electron microscopy, the densities of chromaffin granules per gland (/µm^3^) were assessed for every rat. All samples were assessed by two independent pathologists, who were blinded to the corresponding treatments [29].

### Immunohistochemistry

After being embedded in paraffin at 4°C overnight, tissues were sectioned (4 µm) and mounted on slides. The tissue sections were then deparaffinized in toluene and rehydrated in an ethanol series of increasing concentrations of water. The endogenous peroxidase was blocked with 3% H_2_O_2_ for 10 minutes. The sections were then incubated with polyclonal rabbit antibody against rat (anti-PNMT, AB110, 1∶2500; anti-peripherin, AB1530, 1∶150; both from Millipore, USA) at 4°C overnight, followed by 20 min incubation with biotin labeled goat anti-rabbit immunoglobulin (Ig)G (1∶500; Zhongshan Goldenbridge Biotechnology, China). The sections were then treated with streptavidin-peroxidase reagents (Zhongshan Goldenbridge Biotechnology) and developed with DAB substrate. After counterstaining with hematoxylin, the sections were further incubated with non-immune rabbit sera for use as a negative control. The results of staining were observed under light microscope (100×), and the average optical density was calculated by IPP6.0 software for statistical analysis. The whole field of the adrenal medulla was chosen for every rat and we counted the peripherin-positive ganglion cells under the microscope, and all samples were assessed by two pathologists who were blinded to the corresponding treatments [27].

### Western Blot Analysis

Total proteins were extracted from tissue samples by RIPA Lysis Buffer (Takara, Japan). Thirty micrograms of protein were resolved by 10% sodium dodecyl sulfate-polyacrylamide gel electrophoresis (SDS-PAGE) and electrically transferred to a polyvinylidene fluoride (PVDF) membrane using 120 V for 1.5 hr. The transfer membrane was sealed by incubation with 0.05 g/mL skim milk at room temperature (20°C) for 2 hr. Rabbit anti-rat peripherin polyclonal antibody was added (AB1530, 1∶1000; Millipore) and incubation was carried out at 4°C overnight. After washing with PBST, the membranes were incubated at 37°C for 1 hr with secondary antibodies (1∶5000; Sigma) and visualized by the enhanced chemiluminescence system (Pierce Biotechnology, USA). Band intensities were normalized to that of β-actin, which was used as an internal control. The protein expression in control rats was arbitrarily set as 1.0 to express fold-changes in asthmatic rats.

### Reverse Transcription (RT)-PCR Analysis

Total RNA was extracted tissues using Trizol reagent (Invitrogen, USA), according to the manufacturer’s instructions. RT-PCR amplification of peripherin (137 bp) and GAPDH (307 bp) was carried out with the following primer sequences: peripherin, 5′-GGTGGAGGTAGAGGCAACA-3' (forward) and 5′-TCGGACAGGT CAGCGTATT-3' (reverse); GAPDH, 5′-TGAACGGGAAGCTCACTGG-3′ (forward) and 5′-TCCACCACCCTGTTGCTGTA-3′ (reverse). The thermal cycling program consisted of preheating at 94°C for 5 min, followed by 35 cycles of denaturing (94°C for 30 sec), annealing (60°C for 30 sec) and extension (72°C for 1 min), and a final extension step at 72°C for 10 min. The PCR products were resolved by electrophoresis through a 2% agarose gel in order to confirm product size. Product concentrations were normalized to GAPDH, which was used as an internal control. The gene expression from the control rats was arbitrarily set as 1.0 to express fold-changes detected in the asthmatic rats.

### Enzyme Linked Immunosorbent Assay (ELISA)

EPI, corticosterone and NGF levels in serum were quantified using the ELISA technique. Commercially-available antibodies were applied according to the manufacturer’s recommendations (EPI, 0100-0009, Serotec, USA; corticosterone, 500651-96, Cayman, USA; NGF, BT555, BPB Biomedical, USA). The reactions were measured at 450 nm by using an ELISA reader.

### Statistical Analysis

Data are presented as mean±SD deviation (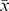
±s). One-way analysis of variance was used for multiple comparisons, followed by the Fisher’s protected least significant difference test. A *P-*value of less than 0.05 was considered statistically significant.
